# Establishment and implementation of an effective rule for the interpretation of computed tomography scans by emergency physicians in blunt trauma

**DOI:** 10.1186/1749-7922-9-40

**Published:** 2014-06-27

**Authors:** Yukihiro Ikegami, Tsuyoshi Suzuki, Chiaki Nemoto, Yasuhiko Tsukada, Arifumi Hasegawa, Jiro Shimada, Choichiro Tase

**Affiliations:** 1 Department of Emergency and Critical Care Medicine, School of Medicine, Fukushima Medical University, 1 Hikarigaoka, Fukushima 960-1295, Japan

**Keywords:** Blunt trauma, Computed tomography, Rule, Misinterpretation

## Abstract

**Introduction:**

Computed tomography (CT) can detect subtle organ injury and is applicable to many body regions. However, its interpretation requires significant skill. In our hospital, emergency physicians (EPs) must interpret emergency CT scans and formulate a plan for managing most trauma cases. CT misinterpretation should be avoided, but we were initially unable to completely accomplish this. In this study, we proposed and implemented a precautionary rule for our EPs to prevent misinterpretation of CT scans in blunt trauma cases.

**Methods:**

We established a simple precautionary rule, which advises EPs to interpret CT scans with particular care when a complicated injury is suspected per the following criteria: 1) unstable physiological condition; 2) suspicion of injuries in multiple regions of the body (e.g., brain injury plus abdominal injury); 3) high energy injury mechanism; and 4) requirement for rapid movement to other rooms for invasive treatment. If a patient meets at least one of these criteria, the EP should exercise the precautions laid out in our newly established rule when interpreting the CT scan. Additionally, our rule specifies that the EP should request real-time interpretation by a radiologist in difficult cases. We compared the accuracy of EPs’ interpretations and resulting patient outcomes in blunt trauma cases before (January 2011, June 2012) and after (July 2012, January 2013) introduction of the rule to evaluate its efficacy.

**Results:**

Before the rule’s introduction, emergency CT was performed 1606 times for 365 patients. We identified 44 cases (2.7%) of minor misinterpretation and 40 (2.5%) of major misinterpretation. After introduction, CT was performed 820 times for 177 patients. We identified 10 cases (1.2%) of minor misinterpretation and two (0.2%) of major misinterpretation. Real-time support by a radiologist was requested 104 times (12.7% of all cases) and was effective in preventing misinterpretation in every case. Our rule decreased both minor and major misinterpretations in a statistically significant manner. In particular, it conspicuously decreased major misinterpretations.

**Conclusion:**

Our rule was easy to practice and effective in preventing EPs from missing major organ injuries. We would like to propose further large-scale multi-center trials to corroborate these results.

## Introduction

In recent years, the use of computed tomography (CT) has enabled rapid and accurate diagnoses in cases of primary trauma [1-5]. CT can be used to detect injuries that are otherwise invisible, but this requires a high level of skill in interpretation. Regular corroboration by a radiologist is therefore necessary to maintain an acceptable level of accurate diagnoses. However, some studies have reported real-time interpretation by a radiologist to be impossible because of a serious shortage of radiologists [6,7]. Additionally, in Japan, emergency physicians (EPs) must currently interpret CT results themselves to decide on a suitable treatment plan in many trauma cases.

Even a slight misdiagnosis may cause death in severe multiple trauma. Most EPs have abundant knowledge of trauma and a high level of skill in primary trauma care, but they cannot provide adequate treatment if they do not correctly identify injured organs. EPs are therefore required to have a high level of skill in interpreting CT results, while knowing that they should always exercise caution in doing so. In our opinion, it is most important to prevent misdiagnosis of traumatic injuries at the first stage of treatment to avoid potentially wrong or unnecessary treatment and any resulting consequences.

In this study, we proposed a precautionary rule to guide our EPs and prevent CT misinterpretation. Through this study, we hope to contribute to the establishment of a safe and effective emergency CT interpretation system for use in blunt trauma patients.

## Materials and methods

Our emergency department (ED) is equipped with a multi-slice CT machine (from Toshiba Medical Systems Corporation) with 64 channels and is always in a state of standby for trauma patients. In blunt trauma, the EP in charge of the ED carries out a primary survey based on a standardized protocol, which actively employs whole body CT. EPs interpret the CT scan at the time of imaging and record their image diagnoses in an electronic clinical chart. From there, the hospital procedure to definitive diagnosis based on CT is as follows. A radiologist interprets the emergency CT obtained in the ED within several hours, and this image report is uploaded to the electronic clinical chart. Every morning, the EPs discuss the radiologist’s report in a trauma conference and then arrive at a final CT diagnosis.

To reduce CT misinterpretation by EPs, we established a simple precautionary rule, which advises EPs to interpret CT scans with particular care when a complicated injury is suspected per the following criteria: 1) unstable physiological condition; 2) suspicion of injuries in multiple regions of the body (e.g., brain injury plus abdominal injury); 3) high energy mechanism of injury; and 4) requirement for rapid movement to other rooms for invasive treatment. If a patient meets at least one of these criteria, the EP should carefully interpret the CT scan. Namely, the EP should undertake the following actions: 1) employment of enhanced CT for chest, abdomen, and pelvis; 2) re-interpretation of the images more than twice after short intervals; 3) changing the window levels according to the organs interpreted; 4) evaluation using not only an axial view but also a sagittal or coronal view when necessary; 5) use of a three-dimensional view to evaluate bone injuries; and 6) repetition of the CT after time has passed.

Additionally, our rule specifies that the EP should request real-time interpretation by a radiologist in difficult cases per the following guidelines: 1) the patient’s physiological condition deteriorates in spite of treatment; 2) laboratory data show the development of anemia or metabolic acidosis in spite of treatment; or 3) unclear points remain in spite of re-interpretation or repetition of the CT. We posted this rule in the CT control room and the ED conference room, and we held a briefing session for our EPs introducing this new rule. We implemented the practice that the EP in charge of the ED must follow the rule. Our precautionary rule is shown in Table 1.

**Table 1:** Precautionary rule for CT interpretation by emergency physicians in blunt trauma

***Caution***	#1 Unstable physiological condition
	1> GCS < 10 points
	2> Systolic pressure < 90 mmHg
	#2 Suspected injury to multiple regions of the body
	1> severe pain in more than 2 of the 6 regions (head, face, neck, chest, abdomen, pelvis)
	2> bleeding, wounds, deformities, or contusions in more than 2 of the 6 regions
	#3 Injury due to high energy mechanism
	1> traffic accident:
	pedestrian, bicycle vs. vehicle, motorcycle crash, highway crash
	victim thrown from vehicle, death of fellow passenger
	case involving a difficult rescue, sideslip of the vehicle, etc.
	2> fall (3 m)
	3> crushed under heavy object
	4> other high energy mechanisms
	#4 Case that requires invasive emergency treatment necessitating movement to other rooms
	1> case that requires an emergency operation
	2> case that requires emergency angiography (embolization)
	3> other invasive treatment required
***Action***	**If patient‘s condition agrees to one of above criteria at least, EP should take action as follows**
	1) EP should actively employ enhanced CT for chest, abdomen and pelvis if possible.
	2) EP should re-interpret emergency CT more than twice after a short interval.
	3) EP should change window level according to organs to interpret.
	4) EP should evaluate not only in an axial view but also in a sagittal view or coronal view if needed.
	5) EP should actively evaluate bone injuries using three-dimensional view.
	6) EP should repeat CT after time has passed if there are unclear points.
***Additional advice***	**If there problems as follows, EP should consider real-time consultation with a radiologist**
	1) Patient’s physiological condition deteriorates in spite of treatments.
	2) Data of laboratory findings show development of anemia or metabolic acidosis in spite of treatments.
	3) Unclear points remain in spite of re-interpretation CT or repetition of CT.

We established a new precautionary rule for the interpretation of emergency CT scans in cases of blunt trauma.

This study comprised two periods. In the first period (before introduction of the rule), the records of CT interpretations in ED blunt trauma cases during January 2011 and June 2012 were reviewed, and the accuracy of the EPs’ interpretations as well as resulting patient outcomes were investigated. In the second period (after introduction of the rule), the accuracy of the EPs’ CT interpretations and the resulting patient outcomes were investigated for July 2012 and January 2013. Finally, we evaluated whether our rule was effective by comparing the accuracy of the EPs’ interpretations and patient outcomes both before and after implementation of the rule.

In both periods, the interpretation accuracy was evaluated by comparing the initial interpretation recorded by the EP and the definitive diagnosis. Each evaluation was independently performed by a senior EP (authorized by the Japanese Association for Acute Medicine) who did not directly participate in the study. When one patient underwent a simultaneous CT scan of several body regions, the results were classified by region and analyzed separately. The evaluation of image diagnoses was performed by dividing the body into the following regions: head, face, neck, chest, abdomen, and pelvis. Checkpoints in each region were evaluated in accordance with the Abbreviated Injury Scale (AIS) (Table 2). In this study, we defined standards for the level of misinterpretation (minor versus major) and the level of gravity (effect on the patient) to evaluate how the level of misinterpretation influenced the clinical course of the patient (namely, we thought that a major misinterpretation, in which an anatomic abnormality was missed, was more likely to lead to a fatal prognosis). Those definitions were designed in accordance with past reports (Table 2) [8-10].

**Table 2:** Checkpoints for the interpretation of each region and definitions

**Checkpoint**	Head	Skull fracture, Basal skull fracture, Brain contusion, Intracranial hemorrhage, Subarachnoid hemorrhage, Subdural hemorrhage, Epidural hemorrhage, Vascular injury
	Face	Bone injury (Ophthalmology wall, Maxilla, Mandible, Zygomatic, Nose), Eyeball injury, Optic nerve injury, Vascular injury (if enhanced)
	Neck	Bone injury (Cervical spine, Spinous process, Transverse process), Pharyngeal injury, Bronchial injury, Vascular injury (if enhanced)
	Chest	Bone injury (Rib, Clavicle, Scapula, Sternum), Thoracic spine injury, Pneumothorax, Hemothorax Pulmonary injury, Bronchial injury, Cardiac injury, Cardiac tamponade, Esophageal injury Diaphragmatic injury, Vascular injury (if enhanced)
	Abdomen	Bone injury (Lumber spine), Parenchymal organ injury (Liver, Gallbladder, Pancreas, Spleen, Kidney, Adrenal gland), Digestive tract injury, Free air, Mesenteric injury, Ureteral injury, Vascular injury (if enhanced)
	Pelvis	Bone injury (Lumber spine, Ilium, Sacrum, Pubis, Ischium, Acetabular cartilage, Femur), Bladder injury, Urinary tract injury, Genital organ injury, Vascular injury (if enhanced)
**Definition of misinterpretation**		
No misinterpretation	All checkpoints were accurately cleared.	
Minor misinterpretation	Anatomical abnormalities were identified, but details were incomplete or incorrect. (e.g., rib fracture was identified but the injured number was misinterpreted; brain injury was pointed out, but the correct diagnosis such as subdural hemorrhage was not recorded.)	
Major misinterpretation	Anatomical abnormality described on CT was apparently missed even if EP received support by radiologist.	
**Gravity level**	The gravity level was determined upon review of the patient’s clinical course.	
	Level 1	Clinical course was not affected by the EP’s interpretation.
	Level 2	Clinical course was affected by the EP’s misinterpretation.
		1) More invasive treatment was required because of the delayed detection of organ injuries.
		2) Temporary functional disorders or persistent cosmetic problems
		3) The course of treatment was unavoidably changed.
		4) Hospital stay was prolonged.
	Level 3	Clinical prognosis was seriously affected by the EP’s misinterpretation.
		1) Permanent, severe functional disorders or cosmetic problems (e.g., persistent disorder of consciousness, limb palsy, large scars)
		2) Death

Checkpoints for each region were established in accordance with the Abbreviated Injury Scale (AIS).

For this study, we used unpaired t-tests for continuous data and chi-squared tests for categorical data, except when the number of expected cells was found to be less than five, in which case we used Fisher’s exact test. IBM SPSS version 21 was employed and all tests were two-tailed, with differences reported as significant for *p* < 0.05. This study was approved by the ethics committee of Fukushima Medical University, and we tried to protect personal information as much as possible.

## Results

In the first period, 365 patients (280 males and 85 females) were identified as blunt trauma patients. Emergency CT was used 1606 times on these patients (361 times for the head, 77 times for the face, 272 times for the neck, 306 times for the chest, 295 times for the abdomen, and 295 times for the pelvic area). The mean patient age was 50.1 ± 23.3 years (expressed as mean ± standard deviation [SD]), and the mean Injury Severity Score (ISS) was 11.9 ± 11.1 (mean ± SD). The cause of trauma was a traffic accident in 186 cases, a fall in 117 cases, and other mechanisms in 62 cases.

The accuracy and outcomes of the EPs’ interpretations from the first period are shown in Table 3. Of the 1606 cases, 44 (2.7%) minor misinterpretations and 40 (2.5%) major misinterpretations were identified. There were no duplicated diagnostic mistakes within an individual case and no pattern of diagnostic mistakes from specific doctors.

**Table 3:** Accuracy and outcomes of EPs’ CT interpretations in the first period

**Region**	**Number**	**Correct interpretation**	**Minor misinterpretation**	**Gravity level**		**Major misinterpretation**	**Gravity level**	
Head	361	338 (93.6%)	15 (4.2%)	1	15	8 (2.2%)	1	7
				2	0		2	1
				3	0		3	0
Face	77	59 (76.6%)	13 (16.9%)	1	12	5 (6.5%)	1	5
				2	1		2	0
				3	0		3	0
Neck	272	267 (982%)	2 (0.7%)	1	2	3 (1.0%)	1	3
				2	0		2	0
				3	0		3	0
Chest	306	281 (91.8%)	6 (2.0%)	1	4	19 (6.2%)	1	14
				2	1		2	4
				3	0		3	1
Abdomen	295	288 (97.6%)	5 (1.7%)	1	5	2 (0.7%)	1	2
				2	0		2	0
				3	0		3	0
Pelvis	295	289 (98.0%)	3 (1.0%)	1	2	3 (1.0%)	1	2
				2	1		2	1
				3	0		3	0
**Total**	**1606**	**1522 (94.8%)**	**44 (2.7%)**	**1**	**40**	**40 (2.5%)**	**1**	**33**
				**2**	**3**		**2**	**6**
				**3**	**0**		**3**	**1**

*Abbreviation*: *EPs* emergency physicians.

Minor misinterpretations occurred in 44 out of 1606 cases (2.7%), and major misinterpretations occurred in 40 cases (2.5%). There were no duplicated diagnostic mistakes within an individual case.

In this period, there were eight major misinterpretations out of 361 cases (2.2%) that underwent head CT (3 subarachnoid hemorrhages, 2 brain contusions, 2 skull fractures, and 1 epidural hemorrhage). One patient judged as a gravity level 2 had a traumatic subarachnoid hemorrhage, brain contusion, and skull fracture detected by the attending EP, but a conscious disorder developed owing to progression of a missed slight epidural hemorrhage. An emergency operation to remove the hemorrhage was successfully performed. Other patients recovered with conservative treatment. There were five major misinterpretations from the 77 cases (6.5%) of orbital plate fractures on face CT, but none of the patients required surgical treatment or experienced persistent functional disorders. There were three major misinterpretations from the 272 cases (1.1%) of spinous process fractures in the cervical spine, but surgical treatment was not required in any.

There were 19 major misinterpretations (6.2%) out of the 306 cases that underwent chest CT (7 costal fractures, 4 transverse process fractures in the thoracic spine, 1 sternum fracture, 1 scapula fracture, 3 pulmonary contusions, 2 cases of pneumothorax, and 1 intercostal artery injury). The patient with intercostal artery trauma did not survive and was categorized as gravity level 3. Three patients with costal fractures and one patient with pneumothorax were categorized as gravity level 2 because a chest drain was required. There were two major misinterpretations from the 295 cases (0.7%) that underwent abdominal CT (1 of liver trauma and 1 of kidney trauma). Neither required any surgical treatment. Anemia did not develop, and both recovered fully without intensive treatment. There were three misinterpretations out of the 295 cases that underwent pelvic CT (1 each for fractures of the pubis, ischium, and neck of the femur). The patient with the femoral neck fracture was operated on by orthopedic surgeons, but the other two patients did not require any surgical treatment. Anemia did not develop in either case, and both recovered fully without intensive treatment.

In the second period, 177 patients presented with blunt trauma, of whom 129 were male and 48 female. In total, emergency CT was used 820 times (171 times for the head, 49 times for the face, 155 times for the neck, 151 times for the chest, 147 times for the abdominal area, and 147 times for the pelvic area). The mean patient age was 50.3 ± 23.4 years (mean ± SD), and the mean ISS was 11.7 ± 9.1 (mean ± SD). There was no statistically significant difference in mean age or ISS compared with the first period. The cause of trauma was a traffic accident in 99 cases, a fall in 44 cases, and other mechanisms in 34 cases.

The accuracy and outcomes of EPs’ interpretation in the second period are shown in Table 4. Of the 820 cases, 10 (1.2%) minor misinterpretations and two (0.2%) major misinterpretations were identified. The improvements between the first and second period were statistically significant. Minor misinterpretations occurred in 2.7% of cases (95% confidence interval, 1.9% to 3.5%) in the first period versus 1.2% of cases (95% confidence interval, 0.5% to 2.0%) in the second period (Fisher’s exact test, *p* = 0.02). For major misinterpretations, the difference was even greater; major misinterpretations occurred in 2.5% of cases (95% confidence interval, 1.7% to 3.3%) in the first period versus 0.2% of cases (95% confidence interval, −0.1% to 0.6%) in the second period (Fisher’s exact test, *p* < 0.01). In the second period, the frequency of minor misinterpretations on face CT was significantly decreased compared with the first period, and there were no minor misinterpretations on pelvic CT in the second period. For head, face, neck, abdomen, and pelvis, there were no major misinterpretations in the second period. For chest CT, two slight costal fractures were missed, but they were categorized as gravity level 1 because they did not require any advanced treatment. In total, real-time radiological support was requested 104 times (12.7% of all cases). In all of these cases, it was difficult to accurately detect injured organs because of complicated trauma, and the additional support meant that effective treatment was carried out.

**Table 4:** Accuracy and outcomes of EPs’ CT interpretations in the second period versus the first period

**Region**	**Number**	**Correct interpretation**	**Minor misinterpretation**	**Gravity level**		**P value**	**Major misinterpretation**	**Gravity level**		**P value**	**Real-time support**
Head	171	169 (98.8%)	2 (1.2%)	1	2	0.07	0	1	0	(−)	17
				2	0			2	0		
				3	0			3	0		
Face	49	47 (95.9%)	2 (4.1%)	1	2	0.03*	0	1	0	(−)	4
				2	0			2	0		
				3	0			3	0		
Neck	155	154 (99.3%)	1 (0.6%)	1	1	0.05	0	1	0	(−)	14
				2	0			2	0		
				3	0			3	0		
Chest	151	146 (96.7%)	3 (2.0%)	1	3	0.38	2(1.3%)	1	2	0.02*	23
				2	0			2	0		
				3	0			3	0		
Abdomen	147	145 (98.7%)	2 (1.3%)	1	2	0.47	0	1	0	(−)	23
				2	0			2	0		
				3	0			3	0		
Pelvis	147	147 (100%)	0	1	0	(−)	0	1	0	(−)	23
				2	0			2	0		
				3	0			3	0		
**Total**	**820**	**808 (98.5%)**	**10 (1.2%)**	**1**	**8**	0.02*	**2 (0.2%)**	**1**	**2**	<0.01*	**104 (12.7%)**
				**2**	**0**			**2**	**0**		
				**3**	**0**			**3**	**0**		

Fisher’s exact test was performed to compare the number of misinterpretations between the first and second periods.

*Indicates a significant difference, with *p* < 0.05. Abbreviation: *EPs* emergency physicians.

In the second period, minor misinterpretations occurred in 10 out of 820 cases (1.2%), and major misinterpretations occurred in 2 out of 820 cases (0.2%). The new rule significantly decreased both minor and major misinterpretations (p < 0.05).

## Discussion

In severe blunt trauma cases, the rapid and accurate detection of injured organs is critical in saving lives. Recently, CT has been reported to be an effective tool for the detection of blunt trauma [3]. In the past, active employment of CT was not recommended because it was thought to expose patients to the risks associated with high levels of radiation [11]. However, CT can detect very subtle organ trauma, and it is applicable to many areas of the body. Nowadays, it does not require the risky long distance transport of severely injured patients because most emergency medical institutions are equipped with highly efficient CT machines. In fact, CT is becoming one of the most indispensable primary examination tools for the diagnosis of acute diseases in addition to its use in trauma cases [12-14].

However, the present interpretation system for CT has not kept up with the modality’s technological development, and real-time interpretation by radiologists is not available in many institutions in Japan because of a nationwide shortage of radiologists. Many EPs, therefore, must make decisions regarding trauma treatment plans without radiological support. Hunter et al. reported that only wet reading was available in the majority of medical institutions surveyed and that emergency CT was usually supported only by radiology residents even in university hospitals [15]. Torreggiani et al. reported that real-time interpretation by radiologists was not available in many institutions and that, in some, radiologist interpretation took more than 48 hours to prepare [16]. They also reported that EPs and radiologists felt very differently about whether the interpretation system was adequate. Many EPs complained of a deficiency in the current interpretation system. Such problems are likely to continue into the long term unless effective measures are taken. Our hope is that this study may provide an effective CT interpretation system for EPs to use in blunt trauma cases.

In this study, EPs misinterpreted 40 of 1606 cases (2.5%) in the first period. Seven of the 365 total patients (1.9%) were most likely placed at a disadvantage by a major misinterpretation; these patients were categorized as gravity level 2 or 3, and they required additional treatments (such as emergency surgery). Chung et al. studied the accuracy of 4768 interpretation reports of torso CT performed by a radiology resident [9]. In this study, serious misdiagnosis occurred in 2.0% of the cases, and changes in treatment were required in 0.3%. Petinaux et al. reported major discrepancies between the interpretations from EPs and radiologists in 3% of cases (for plain chest and abdominal X-rays) [17]. Most of the discrepancies were considered misdiagnoses, and changes in treatment were required in 0.05% of the cases. Gray comprehensively surveyed the occurrence of diagnostic mistakes in the ED [18] and found that 79.7% of mistakes were associated with bone trauma and that most misdiagnoses could likely be avoided by careful interpretation.

There were no large differences in the number and level of diagnostic mistakes between these studies and our study. However, even a small misinterpretation by the EP may lead to irrelevant treatment or a potentially fatal delay in appropriate treatment. This must be avoided wherever possible, but is difficult to achieve in actuality. One solution is to further train EPs to improve their interpretations of CT results. However, a high level of skill is required to interpret CT results, and we believe that it would be almost impossible to improve interpretation ability with unsystematic short-term training. Keijzers et al. evaluated the effect of imaging training in a randomized study and concluded that short-term training did not improve the skill of EPs in interpreting chest CT [19]. The systematic introduction of long-term training would be impossible in our hospital, because EPs are too busy working during the day.

Our study suggested that a simple precautionary rule could significantly decrease misinterpretations without requiring long-term EP training. In particular, the frequency of major misinterpretations decreased in a remarkable manner after implementation of the rule. Our procedure is simple and easy to put into practice, but it proved to be very effective in maximizing the safe interpretation of CT scans by EPs in blunt trauma. Essentially, the rule advised that EPs should interpret emergency CT scans with particular care when a complicated injury was suspected. We believe that the interpretational skill of our EPs is by no means low, but in unstable cases or cases that need invasive emergency treatments, there is a high risk that exact interpretation cannot be carried out. We believe that promoting cautious and meticulous interpretation in every case, but particularly in the cases mentioned above, is effective in preventing misdiagnosis. Our procedure is simple to implement, allowing interpretation to be finished in a short time.

Additionally, our rule specifies that the EP should request the support of real-time interpretation by a radiologist in difficult cases. The interpretations made by a radiologist are not always perfect, but we think that objective evaluation by a professional third party is effective in preventing misinterpretation. We have recently refined our cooperative arrangements, and a radiologist now voluntarily participates in the primary evaluation of major trauma cases. However, success depends on a relatively small group of dedicated radiologists, and it might not be possible to obtain similar cooperation in other medical institutions. Saketkhoo et al. reported that very few radiologists were dedicated to cooperation with the ED [20]. In this study, online interpretation with an electronic chart was used in all cases, which was effective in providing real-time radiology support because radiologists did not have to physically attend the ED. In our study, the incorporation of collaborative real-time support from a radiologist helped to maximize the efficacy of our method.

The problems caused by CT misinterpretation in the ED need to be avoided, and this study represents a first step in establishing an effective and safe CT interpretation system. However, our study has several limitations. First, the number of CT interpretations evaluated was slightly low because our study was conducted in a single medical institute. Second, the definition of the checkpoints may not have been ideal, as severe anatomical injuries were mixed with slight anatomical injuries. Third, the standard for requesting cooperation with a radiologist was not precisely defined. We think that further work is needed to ensure that our method is more widely applicable, and we plan to request cooperation from other critical care centers in Fukushima Prefecture to test it more widely in the future.

## Conclusions

The introduction of a simple precautionary rule, together with collaboration with a radiologist, was effective in improving the accuracy of EPs’ CT interpretations. In the future, we would like to continue these efforts to establish a comprehensive CT interpretation system for blunt trauma patients.

## Abbreviations

AIS: Abbreviated injury scale; CT: Computed tomography; ED: Emergency department; EPs: Emergency physicians; ISS: Injury severity score; SD: Standard deviation.

## Competing interests

The authors declare that they have no competing interests.

## Authors’ contributions

YI designed this study and obtained approval from the ethics committee and cooperation from the radiology department. CT supervised the conduction of the study. TS, CN, and YT managed the data, including quality control. JS and AH provided statistical advice regarding the study design and analyzed the data. YI drafted the manuscript, and all authors contributed substantially to its revision. YI takes responsibility for the study as a whole. Editorial assistance was provided by Edanz, a professional editing company. All authors read and approved the final manuscript.
